# Towards Semantic Integration of Machine Vision Systems to Aid Manufacturing Event Understanding

**DOI:** 10.3390/s21134276

**Published:** 2021-06-22

**Authors:** Kaishu Xia, Clint Saidy, Max Kirkpatrick, Noble Anumbe, Amit Sheth, Ramy Harik

**Affiliations:** 1 McNAIR Center for Aerospace Innovation and Research, Department of Mechanical Engineering, College of Engineering and Computing, University of South Carolina, 1000 Catawba Street, Columbia, SC 29201, USA; kxia@email.sc.edu (K.X.); csaidy@mailbox.sc.edu (C.S.); max.kirkpatrick@siemens.com (M.K.); anumbe@email.sc.edu (N.A.); 2 Siemens Digital Industries Software, Charlotte, NC 28277, USA; 3 Artificial Intelligence Institute, College of Engineering and Computing, University of South Carolina, Columbia, SC 29201, USA; amit@sc.edu

**Keywords:** cyber-physical systems, computer vision, semantic segmentation, cognitive automation

## Abstract

A manufacturing paradigm shift from conventional control pyramids to decentralized, service-oriented, and cyber-physical systems (CPSs) is taking place in today’s 4th industrial revolution. Generally accepted roles and implementation recipes of cyber systems are expected to be standardized in the future of manufacturing industry. The authors intend to develop a novel CPS-enabled control architecture that accommodates: (1) intelligent information systems involving domain knowledge, empirical model, and simulation; (2) fast and secured industrial communication networks; (3) cognitive automation by rapid signal analytics and machine learning (ML) based feature extraction; (4) interoperability between machine and human. Semantic integration of process indicators is fundamental to the success of such implementation. This work proposes an automated semantic integration of data-intensive process signals that is deployable to industrial signal-based control loops. The proposed system rapidly infers manufacturing events from image-based data feeds, and hence triggers process control signals. Two image inference approaches are implemented: cloud-based ML model query and edge-end object shape detection. Depending on use cases and task requirements, these two approaches can be designated with different event detection tasks to provide a comprehensive system self-awareness. Coupled with conventional industrial sensor signals, machine vision system can rapidly understand manufacturing scenes, and feed extracted semantic information to a manufacturing ontology developed by either expert or ML-enabled cyber systems. Moreover, extracted signals are interpreted by Programmable Logical Controllers (PLCs) and field devices for cognitive automation towards fully autonomous industrial systems.

## 1. Introduction

Systematic, rapid, and sustainable artificial intelligence (AI) applications have been underlined to solve production efficiency, product quality, or system reliability related inexplicit manufacturing problems [1]. Recently, the manufacturing world has been intensively developing real-life AI tools for intelligent maintenance, product inspection, virtual metrology, energy management, and scheduling optimizations [2]. Promptly identifying the accumulation of inexplicit problem indicators leads to preventing further explicit deficiencies, such as product quality deviations and machine malfunctions. For instance, taking proactive actions while closely monitoring complex processes, detecting robot precision deterioration, and evaluating system health can ensure progression of manufacturing events, particularly in the context of high precision processes such as assembly, welding, material removal, drilling, and riveting. Therefore, it calls for manufacturer’s attention to closely monitor implicit process events that can lead to seamless changes of operating conditions while silently increase the probability of unpredicted stoppages, by which affect the product quality and production efficiency.

In addition, Industrial AI applications utilize their forecasting capability for business intelligence, such as supply chain demand planning [3], and supplier assessment and selection [4]. Computer-integrated and generative product design are other well-received AI applications in the manufacturing industry [5,6]. Beyond state-of-art AI algorithms, industrial AI applications require reusable system integration, faster solution identification, and more efficient integrations. These industrial use cases have led to the “5S” requirements for industrial smart systems: systematic, standards, streamline, speed, sustainable [1]. Equipped with these requirements, AI-enabled smart manufacturing systems are often implemented with system-embedded closed-loop controls, with cognitive abilities to deliver manufacturing intelligence driven by data and knowledge from devices, processes and, even more importantly, product designs. On a macro scale, future manufacturing industry have been envisioned to address the competition over information technologies [7] provided by intelligent, autonomous, and interoperable industrial systems. It has been concluded that context awareness, interoperability, and compositionality are commonly used to classify a system as a smart manufacturing system [8].

System integration of manufacturing intelligence has therefore been an enabling technology to prepare factories and enterprises for the next evidence-based control and management era, where competitions between enterprises are placed in information technologies. The gaps that need to be filled beyond current experience-based control or management solutions are the system’s capabilities to interpret: (1) process factors not directly available from signals such as images, heatmaps, etc.; (2) the hidden relationships between process factors; (3) the relationship between process factors and quality of process [9]. Moreover, reusability and reconfigurability further indicate the readiness of an AI approach in smart manufacturing systems, namely, the convenience and the compatibility of solving problems on similar devices using the same problem-solving process.

Some pitfalls are identified in today’s data-driven manufacturing AI applications [10]: (1) Focusing on the data science approaches while diverging the core manufacturing problems; (2) Well-established expert knowledge and domain knowledge are not used when understanding, analyzing, and using data, and (3) Humans are not often involved in the data processing and decision making. Thus, the industries desire appropriate adoption of AI techniques that can be enclosed in industrial systems and ready to be deployed into real-time manufacturing applications. Moreover, adequately utilizing domain knowledge instead of pure data science approaches is emphasized. Domain knowledge can include simulated relationships between process factors and process quality, dynamic optimization methods, and/or rule-based expert systems, where semantic integration of high-level knowledge is imperative. Rapid event-understandings by both machine and human in highly complex manufacturing scenes need to be accomplished in this context. This work proposes a novel implementation of vision-based AI control system towards online image inferences for rapid adaptive process control, based on our previous work in image data acquisition [11] and integrated platforms [12].

The rest of this article is organized as follows: Section 2 presents a literature review regarding industrial AI systems. Section 3 revisits the manufacturing cell for our robot assembly line with both digital twin and physical twin setups. In addition, system-embedded image data collection mechanism is introduced. Section 4 presents the authors’ approaches to both cloud-enabled and edge computing towards the event understanding of image feeds. The inference model and results are presented. Section 5 discusses different use cases for these two approaches and proposes a feasible machine vision understanding mechanism combining both pathways. Section 6 concludes this work and anticipates some future research directions based on this work.

## 2. Literature Review

Today, the 4th industrial revolution is replacing the traditional manufacturing control pyramid with cyber-physical system (CPS) enabled autonomous control [13]. CPSs are defined to transfer raw data to actionable operations, assisting users to comprehend and process information, and adding resilience to the manufacturing system through evidence-based decision making [14]. Creation of future manufacturing process monitoring systems requires that the robotics encompassed within the manufacturing process be modeled and connected to their physical counterparts to complete a cyber-physical fusion of data [15,16,17]. The virtual world data from, as an example, simulation-based virtual environment reduces time between development and implementation [18]. Moreover, simulations consist of the ability to replicate various activities within the virtual manufacturing cell and to explore and optimize the actions of the manufacturing process [19]. In addition, development of secured and rapid digital pipelines [20] enables constant back-and-forth sharing of data, which is essentially required for this desired cyber-physical data fusion [21].

The physical manufacturing platform must also enable the collection and integration of process data. In particular, enabling the integration of inspection data with more complete analysis has been studied in some advanced manufacturing processes, such as automated fiber placement [22,23,24]. Traditionally, direct process signals monitored via Programmable Logic Control (PLC) systems, including the control signals indicating the running states of field devices, can be reported over the standardized protocols, such as OPC-UA [25], and MTConnect [26], allowing for easy interconnections to other manufacturing systems [12,27]. The Machine-to-Machine (M2M) communications establish interconnections between machines and autonomous actions over Industrial Internet of Things (IIoT) networks [28]. Benefiting from the convergence of industrial technologies and IP-enabled low-power wireless networking technologies [29], information exchanges between machines can circulate significant process indicators, health indices, along with higher-level manufacturing orders and strategies extracted from external intelligent systems, such as manufacturing feature recognition systems [30], by which means, system adaptiveness given manufacturability analysis [31] can be further explored in the context of complex systems.

In addition, state acquisition for indirect process indicators can be achieved by advanced sensing technologies. Visual systems are proposed as indirect indicators for both process and machine health monitoring [11]. In addition to collection of these high dimensional signals, pattern recognition for time-critical sensing variables have been implemented for continuous event understanding by annotating sensor data with semantic metadata using a semantic sensor Web (SSW) [32]. A specific SSW applied to manufacturing is further developed in [33]. As an instance, in an image-based stream of “manufacturing scenes”, features-of-interests (FoIs), and composite events along with their spatial and temporal attributes [34,35,36] need to be learned. Translating these rich sources of information of “manufacturing scenes” in a way that AI algorithms can understand paves a way to provide rich background and contextual cues to traditional AI algorithms [37].

Industrial AI applications are mostly providing solutions for three categories of research questions: state cognitive assessment, autonomous decision-making (e.g., from “if-then” to “what-if” conditions), and collaborative implementation [9]. Among these questions, AI in signal processing towards smart sensors and online data processing is fundamental as AI algorithms are ideal tools to extract fuzzy logics from complex data to furtherly used in controls, interoperations, or rule-based models [9]. Recent manufacturing technological advances have sought to leverage advanced AI algorithms such as Computer Vision to process images [38,39,40,41], 3D CAD [5,6], and video analysis [42] in industrial scales. Technologies empowering online semantic representation of system states, such as Semantic Sensor Network (SSN) by World Wide Web Consortium (W3C) [43], facilitate the introduction of expert experiences from analysis models, simulation platforms, and more recently, Semantic CPPS [44]. For example, establishing knowledge graphs (KGs) capturing declarative manufacturing knowledge (also referred to as manufacturing ontology) [45,46,47,48] and multimodal data in a machine-readable format for representation, storage, and further reuse [49,50]. Further integration can be expected to adapt communications, such as semantic gateway as service (SGS) [51], in the manufacturing applications.

## 3. Vision System for Process Monitoring

The future of manufacturing is reinventing itself by embracing the opportunities offered by digital transformation, industrial internet, cognitive automation, and artificial intelligence. Cyber-physical systems (CPSs) are looking to pursue the potential convergence of cyber architectures, physical manufacturing processes, and control intelligence. In this section, the authors introduce a novel cyber-physical infrastructure enabled by these technological elements, followed by proposing to utilize a machine vision system to aid general manufacturing event understandings.

### 3.1. Simulation-Based Digital Twin

**Figure 1:**
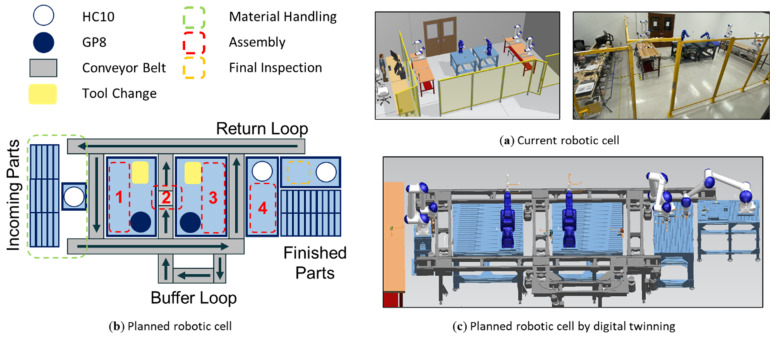
A CPS demonstration at the Future Factories laboratory: (**a**) the current digital twin and physical twin setups; (**b**) robot assembly workflow planning; (**c**) process planning using high-fidelity digital twin system.

This work demonstrates a cyber-physical system of a five-robot assembly line. Collaborative robots from Yaskawa Motoman are controlled by a safety-enabled Siemens S7-1516F PLC system. Industrial sensors and vision systems are embedded as smart devices to monitor the process indicators and device health states during machine operations. The cyber infrastructure is constructed based on a Siemens virtual commissioning solution, Process Simulate, which accommodates a high-fidelity simulation-based digital twin for the physical assembly line. Moreover, programming physical robots within the virtual commissioning platform is not only precise but also intuitive, which does not require a robotic expertise to operate. The automation signals are synchronized and exchanged between PLC and the cyber system via an OPC-UA server. Detailed implementation for this smart manufacturing system was introduced in our previous work [11,12,27]. The overview of this simulation-based digital twin is presented in Figure 1.

Industrial implementations of robotic production lines are widely adopted to automate specific manual processes to further meet the manufacturing requirements in sterility, precision, or workload capacity. However, the needs to adaptively change the robot action sequences in dynamic work cells have drawn the attention of manufacturing practitioners, as expected and unexpected incidents can and do occur during the processes. Such adaptivity requires reliable, precise, and prompt manufacturing event-understanding by machines. Hence, this work proposes to develop a deployable vision system connecting the cyber and physical world. The synchronized results from multiple vision sources, such as inspection cameras, thermal cameras, and unmanned drones, are expected to aid the machine event-understandings along with the signals from conventional industrial sensors. State-of-the-art computer vision algorithms, coupled with manufacturing feature recognition systems [30] and reinforcement learning [12], are hereby supporting real-time autonomous decision-making in the cyber-physical production system. Based on these technological advances, future manufacturing will expand the current research on human–machine interoperability to industrial paradigms, towards cloud-based, service-oriented cyber manufacturing architectures [7] to enable the communications and collaborations among the machines, between human and machines, and among humans.

**Figure 2:**
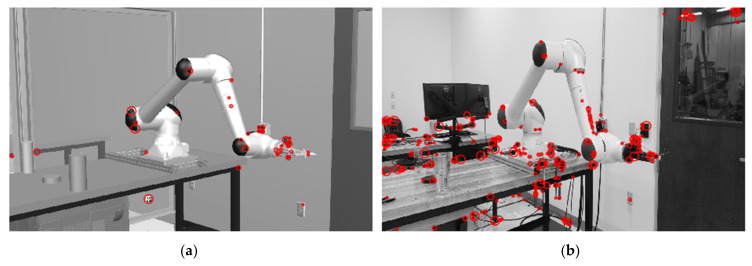
CenSurE [52] features on: (**a**) a virtual image generated by the digital twin; (**b**) a real image from the physical cell.

**Figure 3:**
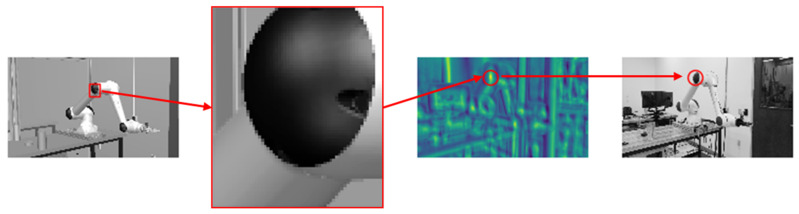
Template Matching [53] to convolute a virtual image feature on a physical image.

Naturally, the vision data samples acquired from this virtual-physical system can be divided into two datasets with the virtual cell images and the physical cell images. Visual recognition accomplished on the physical cell dataset is the ultimate goal to pursue the convergence between virtual and physical world. However, compared to the real-world images, the virtual images are cleaner and significantly easier to draw conclusions from, while containing the features of interest aligned with the physical images (Figure 2). More importantly, manual labeling the physical images can be a cumbersome task in supervised learning tasks. Aided by the digital twin platform, the images are automatically labelled (discussed in Section 3.2) and can be generated with an unlimited number of cameras, robot positions, and process events, etc. Demonstrated similarity of virtual and physical images can largely augment the physical datasets (Figure 3) and start enlightening Computer Vision algorithms with all virtual scenes with automatic labelling.

### 3.2. Data Collection

Vision-based tools have been developed to access the system data from manufacturing processes and devices by displaying the states of system components and the statistics of embedded sensor signals. As an example, Augmented Reality (AR), as a data visualization tool, is utilized in human-centered mechanical assembly lines where real time vision recognition and CAD projections are implemented to instruct practitioners [54]. The goal of these dynamic vision tools is to have a better real-time representation of the system’s states and to be able to act proactively and allow users to reduce audit times.

**Figure 4:**
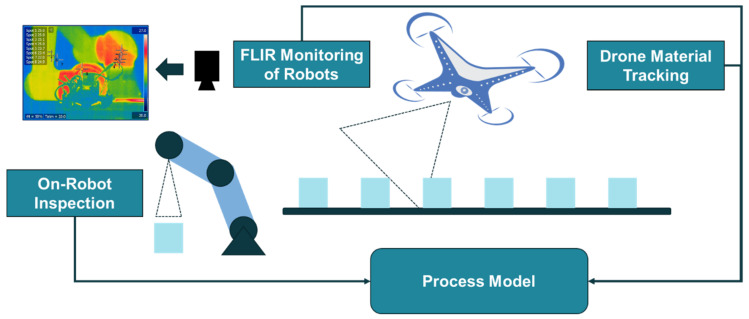
Vision-aided process/health monitoring system for proposed robotic assembly line.

The design of our vision system for process and health monitoring in a robot assembly line is introduced in [11]. The proposed visual system is composed of high-resolution security cameras, wireless on-robot inspection cameras, material tracking drones and infrared thermal cameras (Figure 4). This work attempts to develop an automated semantic processing system to aid machine or human to monitor and assess the conditions of the line by analyzing vision data from multiple ends, and to couple this data with live video feeds. Vision data collected through single-board computing devices will be acquired in real time with minimal latency. Depending on the data source and use cases, collected data are either transferred to a cloud server or analyzed at the edge. The cyber infrastructure will receive, process, and distribute the inference results from the cloud or the edge for a synchronized semantic integration in the control loops. Semantic integration of the vision system provides interoperable signals in an organized manner while ensuring all vital information is readily available for a human user or a machine end.

**Figure 5:**
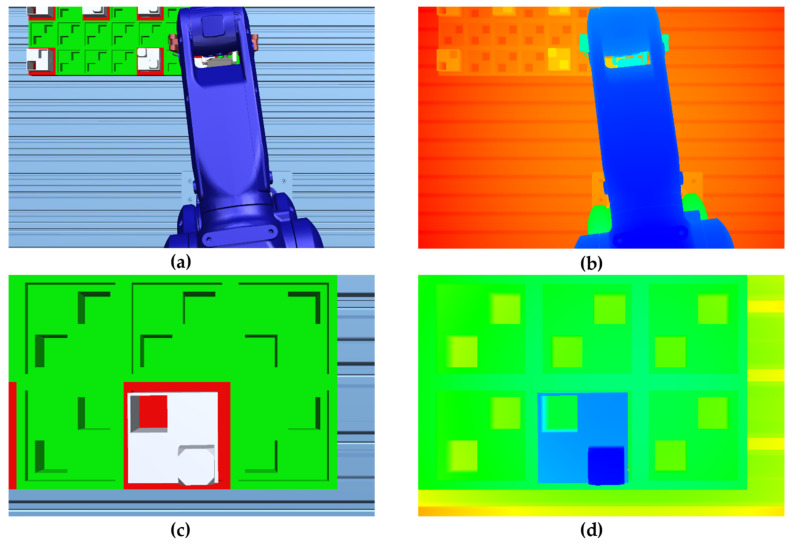
An automatic image annotation method with non-deviation in object shape enabled by our digital twin platform. (**a**) A virtual scene containing robot work in progress rendered by CAD and process simulations; (**b**) The RGBD image used to filter out the mask for the robot; (**c**) A virtual scene containing manufactured part; (**d**) The RGBD image with the same view containing part.

Manual labeling a large image dataset is cumbersome and can be prone to errors, especially when annotating irregular shapes for robots, part defects, etc. To solve this issue and aim to accelerate the image annotation process, a novel method to use digital twin to automatically label object shapes is enabled by our digital twin platform. We use Siemens Tecnomatix Process Simulate to build our digital twin system coupled with CAD models and process simulations. This digital twin implantation has been proved to be highly synchronized and precisely aligned with the real-life processes [12]. Inspired by the discussion of image feature mapping in Figure 3, we expand this digital twin’s usage to annotate object shapes automatically during the process simulations by setting up a virtual vision camera and a RGBD camera in an identical view. The depth information in the RGBD images can be used to filter the vision images shape for masks of desired objects. In Figure 5, it is presented how to use the depth information to filter out the shapes of the robot and the part. This automatic annotation method is not only without any deviation in shape, but also automatically label the object type. Such usage of digital twin can also be extended to any computer-aided solutions that supports image rendering and graphic information retrieval. The virtual images carrying the important features can be envisioned to accelerate the learning process on the physical images, which will be discussed in the following sections. Moreover, monitoring manufacturing processes using RGBD cameras is becoming a more generally accepted industrial practice. Hence, coupled with a real RGBD camera, our image annotation methodology can be directly applied to physical images to filter, detect, and recognize feature of interests in real manufacturing scenes.

## 4. Results

Precise understanding of a manufacturing scene requires analyzing feeds from multiple cameras (security cameras, inspection cameras, drones, thermal cameras, etc.) as well as learning the “manufacturing context” as defined by the other sensor data, participating objects, and sub-processes. Acquiring this knowledge in a generalized and transferable form aids in properly understanding the underlying manufacturing event. A computationally feasible intelligent system embedding by distributing computing powers has been commonly approached [2] by two stages: (1) signal processing and expert feature extraction at the edge or near the source of data, and (2) data-driven modeling and cumulative learning on the server (local or cloud). Semantic integration methods of this proposed vision system are discussed in this section with two implementations: (1) cloud-based service-oriented object recognition; (2) customizable local shape recognition. A novel method of image dataset augmentation is also proposed by the aid of the constructed digital twin setup.

### 4.1. Cloud-Based Object Detection

Beyond the automation pyramid proposed by ISA-95 [55], RAMI 4.0 [56], recent manufacturing paradigms for the integration of enterprise and control systems are decomposing to networked distributed services. For example, NIST service-oriented Smart Manufacturing architecture [57] proposed the utilization of a manufacturing service bus to combine different services, such as modeling and simulation (enterprise digital factory or digital twin) services, business intelligence, and computing/control ends (real factory), by which means, the business intelligence developed as a cloud service can be deployed to each of the manufacturing processes. Enabled by cloud services, the service-oriented architectures (SOA) become commercially deployable. IBM I4.0 proposed a 2-layer decentralized manufacturing system architecture: hybrid cloud layer and device layer. In this work, image uploading and result query using Watson™ IoT platform over the IBM cloud™ are enabled by representational state transfer API (RestAPI) to extend factory’s computing capability. Data are further utilized across various levels: edge, plant, and enterprise, facilitated by distributed computing power from the cloud [58].

IBM cloud™ is a set of cloud-based products for a wide range of IT applications, including database management, AI development, computing servers, IoT platforms, etc. [59]. It provides an environment that helps simplify data preparation processes and model building operations using a set of tools and machine/deep learning capabilities in the cloud. This work explores an AI development use case using Watson Studio™ and presents the system integration process, including image result queries and systematic deployment. Other products will be further explored in future work.

**Figure 6:**
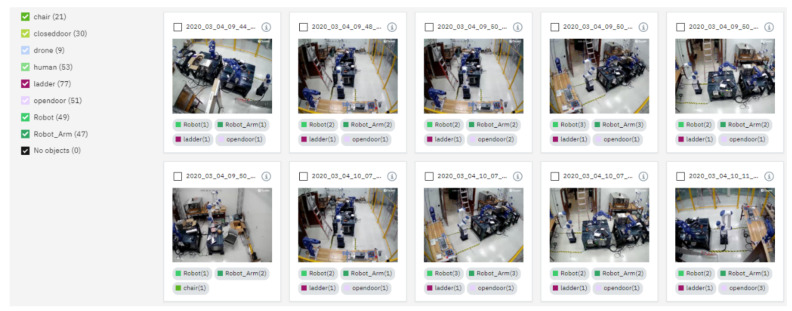
Define object classes for detection in Watson Studio™.

**Figure 7:**
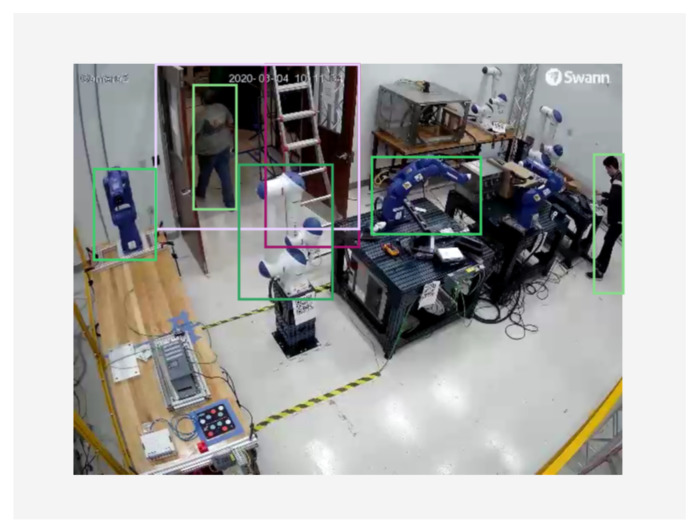
Region annotation by bounding boxes in Watson Studio™.

**Figure 8:**
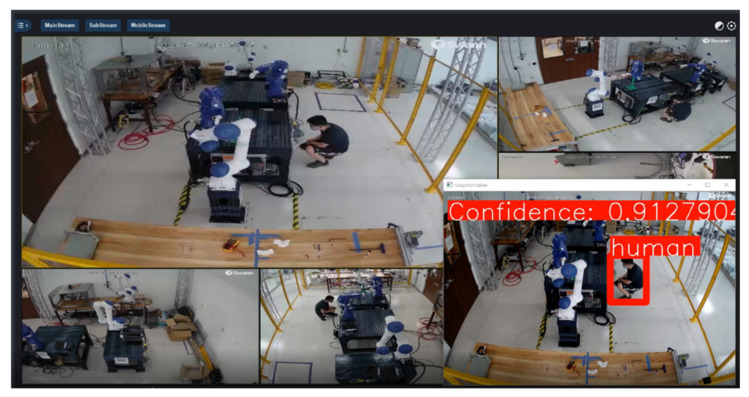
Integrating visual recognition into the cell for an alien object detection task.

Training deep learning models by Watson Studio™ is intuitive, simply by uploading the images and labelling them using web-based interfaces, shown in Figure 6 and Figure 7. The embedded cloud computing power trains the images or detects test images for regions of interest shaped by bounding boxes. Each derived model is designated with an API endpoint, which is used to query the model. Knowledge from the trained model is used to infer a result from uploaded images. The query results return a JSON file with a list of detected regions and their detection confidence scores. The authors embedded cloud-based object detection model in the monitoring devices by scripting the image query pipeline with URL syntax using Client URL (cURL) [60]. A near-synchronized alien object detection result fed by IP security cameras is shown in Figure 8.

Computer vision algorithms are taught by feeding various examples of images already tagged with the needed contents to be identified by the model. Appropriate ratios of both positive and negative image sets are used for training the algorithm. In the case below, we notice an open door that is being annotated, a negative case would be to train the model with images where the door is closed.

Other than the feature of classifying images from our cell, another capability provided by Watson Studio, that we are expanding on, is object localization. Region-Based Convolutional Neural Networks (R-CNNs) [61] have been traditionally used for handling object localization. This capacity would help the operator better understand and locate undesired objects inside the cell. Localization finds a specific object’s location within an image and displays the results as a bounding box around the detected object. The main challenge that arises with the use of this feature is the boundary identification problem that arises when an overlap of two or more objects occurs in an image. To remedy this problem, we are working on a solution that involves analyzing and mapping feeds from different views.

### 4.2. Edge End Object Detection with Shape Recognition

Driven by recent emerging architectures [56,57,58,59], there is an upcoming shift of manufacturing paradigms towards service-oriented AI-integrated platforms, with IBM Watson™ being one of the off-the-shelf commercial products. However, there are still gaps between generally applicable manufacturing processes and cutting-edge AI successes [62], anticipated by self-cognitive and self-configurable cyber-physical systems [63]. Most manufacturing actuations require high precision for real-time location and detection techniques for shape-critical objects. Especially in the material handling processes, such as the feature related assembly [64], automated feature recognition [65] and additive manufacturing path planning [66], recognizing patterns in visual signals requires result reproducibility, acceptable data qualities, knowledge representation design and labeling, and system result reliability assessment. These outstanding manufacturing AI challenges are faced by computer vision scientists whose scope is outside the system engineering and manufacturing domain.

This work attempts to implement a system integration of a recent Computer Vision algorithm in the context of manufacturing processes with object shapes being recognized and output as binary masks. In the Computer Vision community, Convolution Neural Networks (CNNs) [67] have generally been recognized as some of the most effective tools for visual recognition tasks. From LeNet-5 [68], AlexNet [69], VGG-16 [70], to ResNet [71], computer vision scientists have delivered numerous optimal CNN architectures to successfully solve machine learning problems such as overfitting and vanishing or exploding gradients. Built on these classic architectures as backbones, region-based CNNs (R-CNNs) [61] become a category of networks designed for image region classifications and segmentation for object detection tasks. R-CNN uses selective search to generate regions with an elongated inference time. To improve this, Fast R-CNN [72] generates feature maps for selective search algorithm and greatly reduces the required computation times. Faster R-CNN [73] replaces the selective search with Region Proposal Networks (RPN) and further accelerates processing time towards real-time object detection tasks. Using this methodology, Mask R-CNN [74] adds an extra output as the object mask with a small computational expense. As a result, Mask R-CNN performs three tasks: classification, regression, and segmentation, then successfully refines the bounding box and generates a mask in pixel level of the recognized object. This specific segmentation step relates sematic results with object shapes, and hence reduces the object image to a binary shape information. Therefore, this work selects Mask R-CNN as the semantic segmentation algorithm for three reasons: (1) low computational costs provided by Faster R-CNN; (2) Shape-critical recognition results; (3) Binary representation of objects as process event understanding signals. The Mask R-CNN is an extension of Faster R-CNN by constructing a mask branch and implementing a pixel-to-pixel alignment between inputs and outputs. This enables a spatial quantization for feature extraction using binary masks detected from images. A fast train and test speeds of object detection by bounding box are also demonstrated by 0.2 s per frame (5 fps) on a GPU [74]. The improvement of computation comes from its parent network Faster R-CNN [73], which implemented a framework of RPN and has demonstrated the highest 17 fps with the ZF net and 5 fps with VGG net. Compared to test speeds of R-CNN (49 s per image) and Fast R-CNN with object proposal time (2.3 s per image), Faster R-CNN and Mask R-CNN have nearer real-time image processing rates.

**Figure 9:**
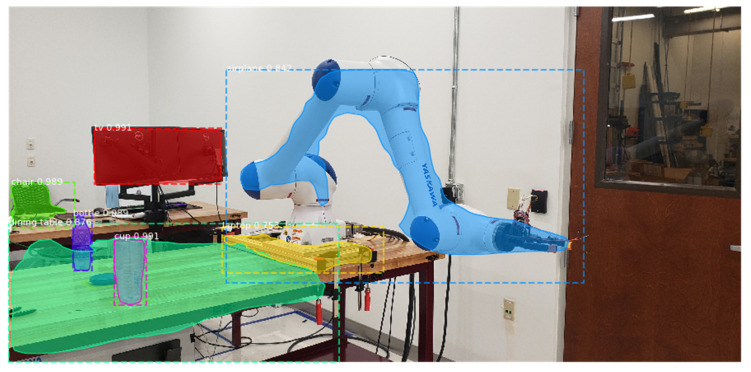
Detecting robotic manufacturing scene using pretrained Mask R-CNN model on COCO dataset as the starting point.

Transfer learning [TL] is another important approach in the machine learning field. Transfer learning intends to leverage prior trained models to resolve more specific, unexplored, and more complicated datasets instead of training models from scratch. TL is highly facilitated by reusing pretrained neural networks as they successfully learned useful features in encountered datasets. Normally, training large networks such as ResNets from scratch requires enormous datasets for convergent results. This is caused by a large number of parameters for wide-ranging image recognition tasks, e.g., ResNet-50 has over 23 million trainable parameters. In this work, pretrained Mask R-CNN weights [75] on a Common Objects in Context (COCO) dataset [76] is set as the starting point to train our dataset. Although pretrained model can recognize 80 classes of COCO object types, including human, plane, table, etc., we still need to define additional classes to understand a robot assembly process. In a robotic manufacturing scene (Figure 9), pre-trained Mask RCNN model on COCO is already able to correctly detect defined objects such as the tables, cups, screens, bottles, and chairs. However, manufacturing assets that are not pre-defined by COCO dataset need to be defined based on the application. In this scene, the shapes of robot fixture and robot arm are successfully detected with incorrectly recognized object classes. Thus, our work based on this pretrained model needs to define indicative manufacturing object classes for event understanding tasks, including the robots, alignments, parts, and fixtures. Any other types of Feature-of-Interest can be further defined should they make contributions to process understanding, such as manufacture features of parts, robot joints, gripper fingers, or the conveying systems, etc. Higher resolution of object class definition will facilitate the understanding of process, meanwhile requires increased samples to adequately train the existing model, especially for uncommon object classes such as customized parts and grippers.

**Figure 10:**
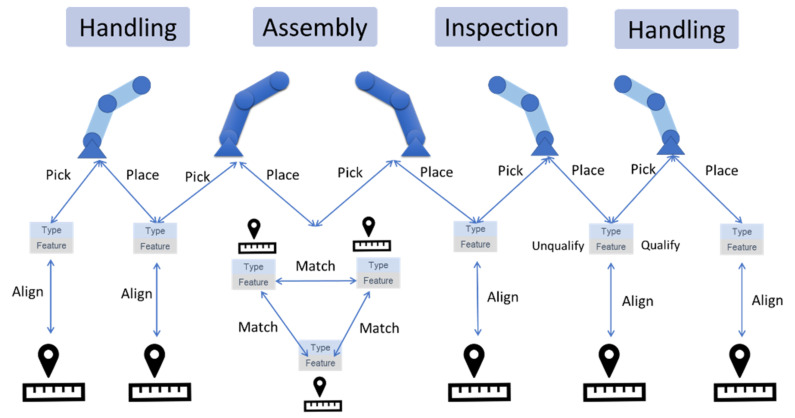
Object classes and manufacturing events defined in the proposed robotic assembly line.

To address the aforementioned challenge of defining customized shapes and ambiguous object classes, the authors also propose to accelerate the training by data augmentation and learning transferred from the virtual datasets. In a manufacturing scene recognition task, when customized objects and shapes classes are beyond numeration, rendering images from virtual environment can accelerate the convergence of training process by virtually simulating FoIs from different angles. Furthermore, utilizing the depth information calculated in the virtual environment will automatically detect object location and hence to label the images without human intervention. The detailed approach and validation are shown in Section 3.1. However, this approach is beyond the scope of this work and will be further discussed in our subsequent work. To aid the visual understanding of the robot assembly events, the physical dataset is labelled by three classes of objects: robot, part, and alignment. For the robots, two robot models (GP8 or HC10) are defined as their attributes. The part types are labelled by the manufacturing tasks, for example, task 1 as the part stacking task and task 2 as the rocket assembly task. Part attributes are labelled with the manufacturing features on them, such as extrusions, blind holes, through holes, etc. The alignment is defined as the marker on the workstations to align parts and instruct the robot where to pick or place parts. The manufacturing events can be interpreted as the linkage between detected objects, which can be defined as pick, place, align, match, part qualify, or unqualify. These robot assembly events were proposed to be understood by the industrial sensors [11], aided by vision systems developed in this work. A graphic representation is shown in Figure 10.

**Figure 11:**
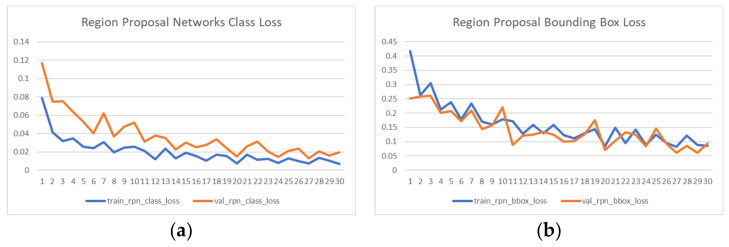

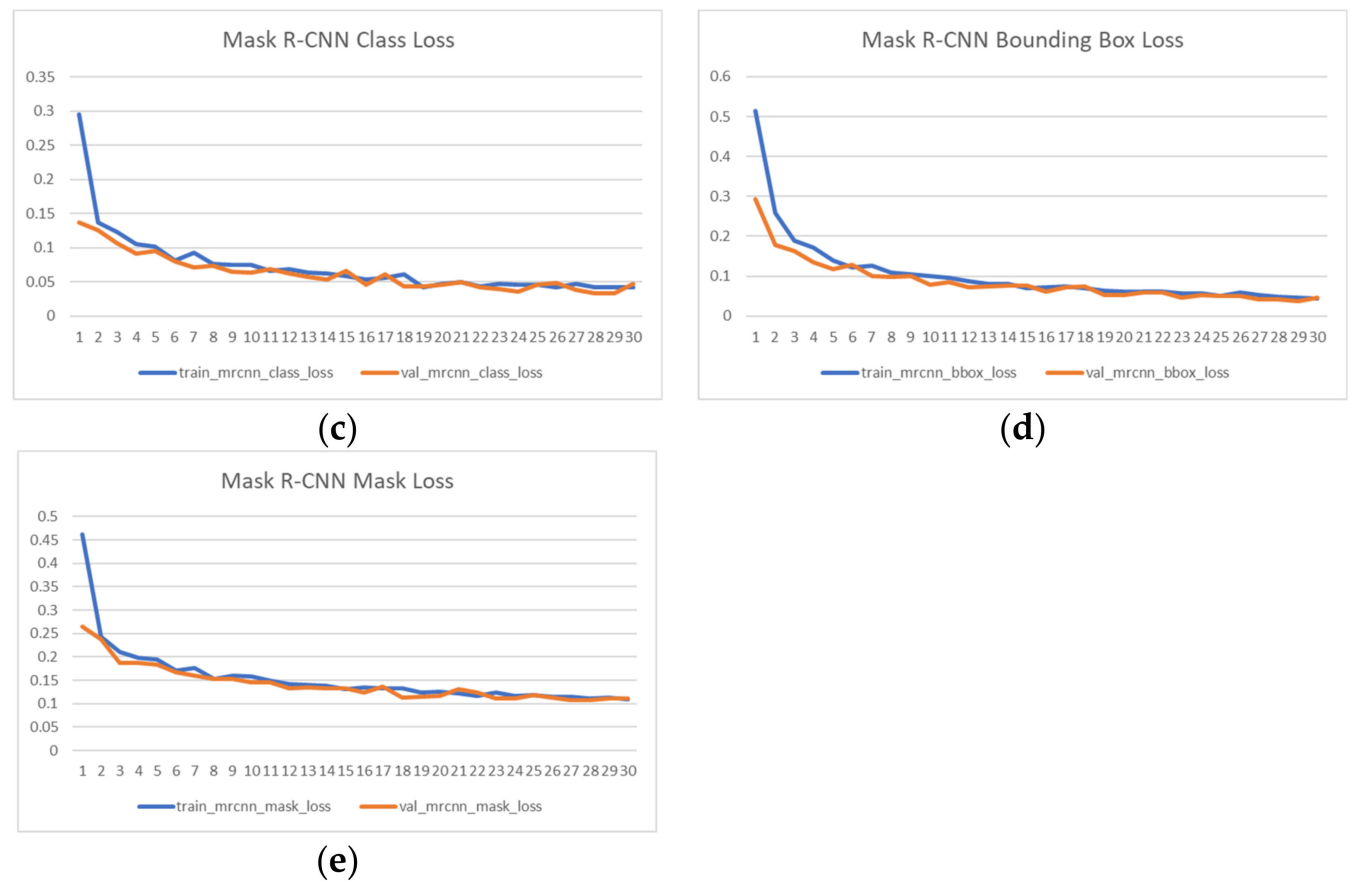
Training and cross-validation curves recorded by 5 loss metrics: (**a**) Region Proposal Networks Class Loss; (**b**) Region Proposal Bounding Box Loss; (**c**) Mask R-CNN Class Loss; (**d**) Mask R-CNN Bounding Box Loss; (**e**) Mask R-CNN Mask Loss.

The object network is trained by a physical dataset with 479 images collected from 10 different camera scenes in our assembly line. The training is performed by 30 epochs and at the end of each epoch, the training losses and random subsampling cross-validation losses are recorded by 5 metrics: (a) Region Proposal Networks Class Loss; (b) Region Proposal Bounding Box Loss; (c) Mask R-CNN Class Loss; (d) Mask R-CNN Bounding Box Loss; (e) Mask R-CNN Mask Loss. The cross-validation is performed 50 steps every 100 steps of training at each epoch. We chose to sample larger validation subsets over traditional k-fold cross-validation because more accurate validation statistics will better determine the performance for adequately pretrained models. That is to say, the actual training task is greatly alleviated by pretraining on the COCO dataset. Performing larger training portion such as 5-fold and 10-fold cross-validation might bring unnecessary training steps as well as the problem as over-fitting. The learning curves are presented in Figure 11. It can be observed that even though the image count for our available dataset is relatively small, the training and validation still converge quickly, as we based our learning on pretrained models with partial recognition capabilities of some shared image features.

**Figure 12:**
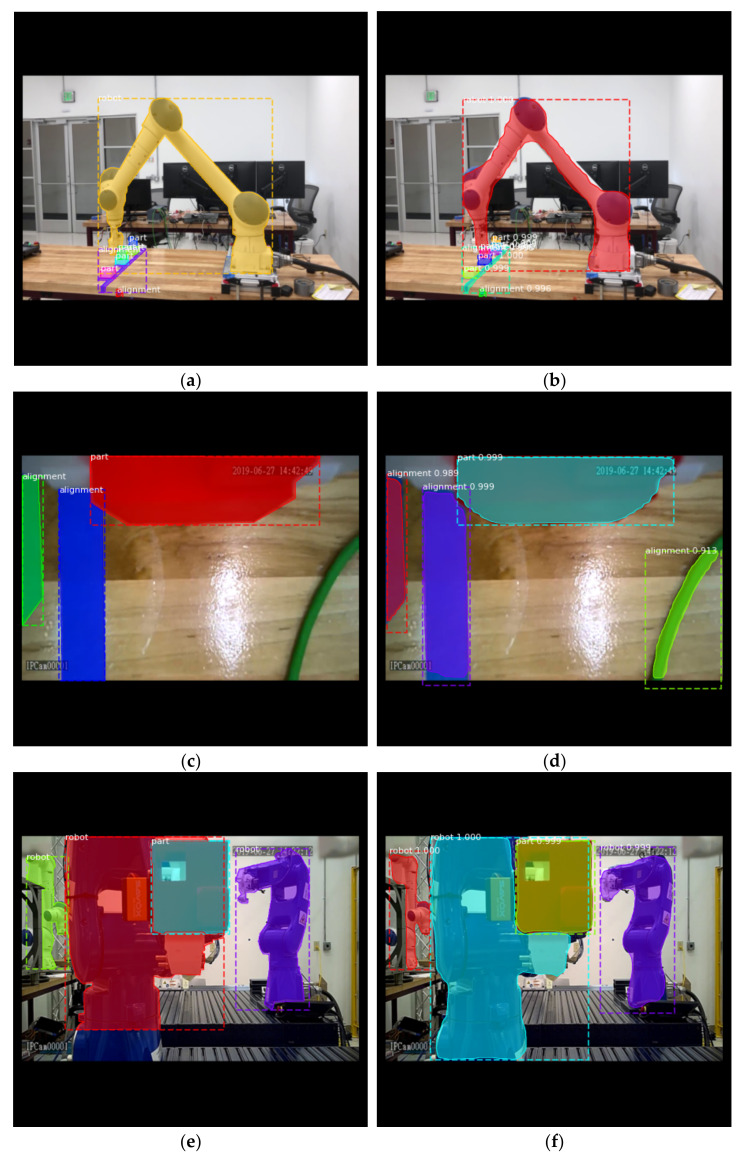
In robot assembly scenes, Mask R-CNN visual detection from multiple monitoring devices: (**a**) Ground truth from ambient camera; (**b**) Inference results from ambient camera; (**c**) Ground truth from inspection camera; (**d**) Inference results from inspection camera with a false positive case; (**e**) Incomplete annotated ground truth from inspection camera; (**f**) Inference results of the incompletely annotated image.

The trained model is able to detect most defined objects and recognize their classes in different scenes, with their shape masks output in binary formats. Some inference results compared with manually labeled ground truths are presented in Figure 12. Each detection is output with a score to represent how confident the recognized result is. One should be aware of the fact that machine learning algorithms are based on statistical evidence instead of deterministic results, such as simulations, rule-based systems, or empirical models. This means the recognized objects are classified by different categories with the corresponding likelihoods, which are represented by the confidence scores. The learning models may be confused by ambiguous inputs and derive wrong results. As an example, in Figure 12d, our model infers the green wire as a blue alignment line, which is a false-positive case. This can be easily understood with human vision that the blue alignment lines and the wire are similar in shape, and they appear in the same background of the wooden desktop. Even so, the model still detects a subtle difference in their color, which is reflected by a significant lower confidence score (0.913 compared to over 0.99 in true positive cases). Such human observable knowledges are important to ensure that we are obtaining the most precise results possible beyond machine learning systems. Thus, another layer of expert system is hence needed in these cases to facilitate a human–machine knowledge fusion. In this specific case, to filter detected objects by thresholding on their confidence scores and then on their color codes can be an obvious solution. This practice of human–machine interoperability is exactly where semantic integration plays an enabling role in manufacturing systems.

Another issue to pursue with supervised learning by Mask R-CNN is that the manual shape annotation process is often performed with errors and deficiencies, as shown in Figure 12e. The base of the robot is not perfectly annotated within the shape region. However, the regression capability of machine learning algorithms manages to alleviate this small deviation by learning from other correctly annotated robot regions, and still infers the shape in a high precision (Figure 12f). As introduced in Section 3.2, we can also choose to use an automatic image annotation method to automatically process the image data. This practice will be further adopted in our subsequent research efforts.

**Figure 13:**
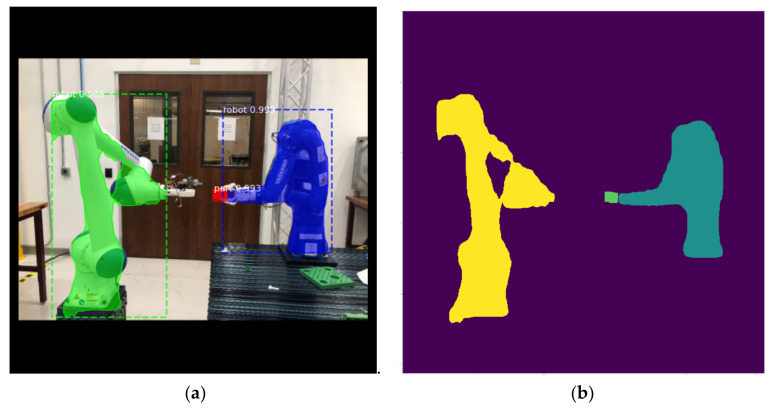
Test Mask R-CNN inference results: (**a**) detection results from a new assembly scene; (**b**) extracted masks for detected objects.

Trained Mask R-CNN model is tested with new video feeds in which both the camera positions and robot operations were not included in the training or validation datasets. In this new scene presented in Figure 13a, derived model successfully detects object shapes and locations with confidence, based on these results, a mask only representation of this manufacturing scene is extracted and can be clustered into a region adjacency matrix storing connectivity information between regions. This temporal information is directly mapped to signals in the control loop administrated by physical PLC devices and OPC-UA server for semantic process monitoring or interoperation. Note that the test result set can be relatively small when validation statistics are being monitored, this is due to the fact that under fixed manufacturing scenes the vision feeds are usually similar, hence the cross-validation measurement is adequate to assess trained models. To summarize, the interpolation capability is more desired over extrapolation capability for our model.

### 4.3. Control Decentralization in Future Factories

Decentralized control architecture has been an on-going research topic in smart manufacturing systems. Future of industry is envisioned to be built on collaborative networks [7] which facilitate flexibility in product customization, product quality control, process control, and service delivery. Human-involved interactions and information flow among machine, people, organizations, and societies towards the next-generation manufacturing paradigm need to be generally adopted among entities. Hence, the capability of semantic process representation becomes an enabler to extract highly complex process data from advanced sensors as signals that can be understood by machine.

In our previous work, we implemented a CPS for factories that automates process self-optimization using Deep Reinforcement Learning based AI. The concept is illustrated in [12]. The control network administrates digital and physical twins, Human–Machine Interface (HMI), and smart devices to synchronize signals and intelligently actuate field devices. Based on this concept, this work proposes to develop semantic representation from images and videos, hence to automate manufacturing intelligence derived by artificial intelligence or established expert knowledge.

**Figure 14:**
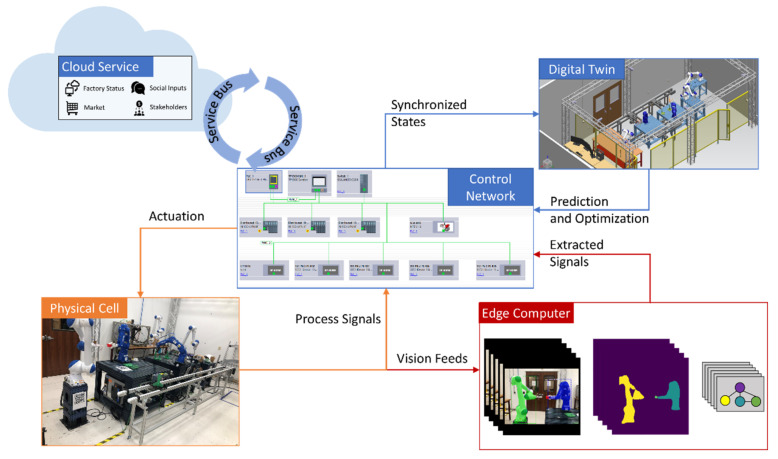
Proposed CPS-enabled control for future factories. Control network administrates physical cell and digital twin to synchronize process signals and intelligently actuate field devices by system smart layers. System smart layers consist of business intelligence from cloud services and semantic integration of visual signals from the edge ends.

Profinet, as a newer and preferred industrial communications protocol, constructs a control network for the robot assembly factory, see Figure 14. This factory consists of field devices, namely robots, tools, and conveyors; controllers, namely PLC, distributed control modules, HMIs, and Ethernet switches; smart devices, namely the sensors, cameras, scanners and edge-end computers; and safety-related devices. For large scale factories, it is needed, and sometimes required, to decentralize control modules and computing powers among the cell. Therefore, the authors implement a decentralized control architecture to build an efficient system-level data layer to exchange signals, images, videos, or 3D data. The control architecture (Figure 14) consists of 4 major components: physical cell, digital twin, cloud service, and the edge-end computing engine. These components are assigned with different tasks. Data collected from the (1) physical cell are transmitted to: (2) the cloud-end for business related big data analytics; (3) the digital twin for fast simulation-based status prediction and fast optimization; (4) edge computing for fast device status extraction. All of these data transmission must be reported and administrated by control network hosted by OPC-UA server in PLC devices. Derived optimal action can be pushed to either the digital twin for virtual commissioning [27] or the physical cell for deployment. In fact, this high-fidelity simulation-based digital twin [12] adds another layer of expert knowledge and substantially increases the reliability of data-driven approaches adopted in the control loop.

Cyber security management has been argued as one of the pillars in future manufacturing systems. To decrease latency while improving energy, efficiency, and security, processing data near or at the source of data has been highly demanded along with the cloud AI applications [77]. From the cloud services, information is exchanged over an enterprise service bus (EBS). In a service-oriented architecture (SOA), an EBS is essential as it connects the legacy system to cloud services after user authentication and authorization. However, as such connections break the sub-system isolation, data security and privacy become a major concern. To that end, another promising perspective of this work is to provide data protection by enclosing raw data processing at the edge level within the monitoring of wired local area networks, while Secure RestAPI sends selected messages via HTTPS requests with Transport Layer Security (TLS) encryption. The local edge computers are connected using Industrial Ethernet protocols, e.g., Profinet. Connected computers are securely accessed through Secure Shell Protocol (SSH) to operate services if over an unsecured network. The lack of security research work in modern smart manufacturing systems is addressed in [78], which includes the topics of testbeds and simulators, attack generation and intrusion detection, forensics, and security policy specification/enforcement. The authors will further investigate these topics for the proposed system in future work. 

## 5. Discussion

This work adopts two approaches to vision-based manufacturing event understanding: (1) a cloud-based service through RestAPI, and (2) edge-end object recognition using Mask R-CNN. Training on IBM cloud services is substantially easier to use and few data science knowledge required, as manufacture practitioners do not need to understand and choose the right learning algorithm. Moreover, the computing power is greatly enhanced if training on the server cloud. However, a significant latency for real-time inferences remains in the image uploading and result querying actions, as the functionality of cloud-based measures depends heavily on communication networks. Meanwhile, service dependable AI solutions give limited options for customized use cases, such as shape recognition and manufacturing feature localization. To address these concerns, the authors tested trained Mask R-CNN model locally supported by a TITAN-V GPU card. Influenced by image resolutions and information carried by regions of interests (RoIs), the processing time for different images varies slightly. Running 10 experiments with randomly selected image feeds, an expectation of averaged processing time of 0.4 s per frame of image is obtained and 9 out of these 10 runs get mean average precision (mAP) of over 0.95 (as shown in Table 1), which validates the potentials of Mask R-CNN as a promising solution for near real-time feature recognition and online tracking in manufacturing scenes. We use VOC-Style (ranging from 0 to 1) mAP at an Intersection over Union (IoU) of 0.5, which measures, averaged by object classes, the averaged maximum precision after each step of reaching to a correct detection with detected object and ground truth pair of overlapped ratio over 0.5. In multi-class and multi-region object detection tasks, mAP is a well-received and efficient metric to assess the performance of algorithms.

**Figure 15:**
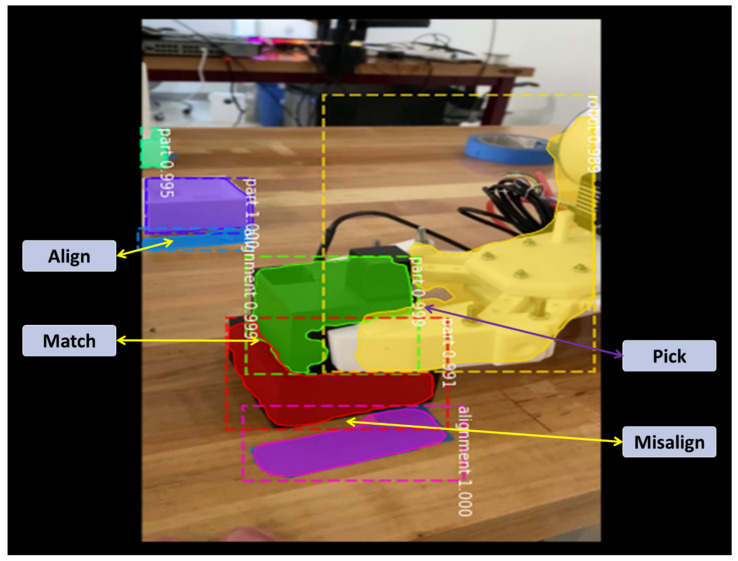
Towards a manufacturing ontology with machine-readable semantic integration of real-time scenes.

Based on these observations, the authors propose to adopt these two approaches accordingly for different visual inputs. In our implementation, using security camera for alien object detection can be designated to the model in server cloud. The images from the security cameras intend to observe and monitor the environmental changes, which means a wide range of objects regularly reside in the visual inputs, such as robots, tables, chairs, ladders, doors, etc. (see Figure 8). Processing images with large amount of RoIs require larger computing powers. In addition, compared to real-time process monitoring, environmental changes in a typical work cell are usually less time critical. Hence, dynamic process changes with shape recognition capabilities within the work cell are proposed to be monitored and detected locally by the feed of inspection cameras, FLIR cameras, and drone cameras. The processing power is preferably distributed near the data source at the edge devices [77] to further reduce the latency. An event-understanding system combined with ontological representation of manufacturing processes can be hereby described by the segmentation information and regional relationships among the binary masks, shown in Figure 15. Integrated with other process signals, the semantic information can be synchronized within the conventional signal-driven control programs running in industrial field devices.

Depending on different manufacturing tasks and applications, the method towards semantic integration varies. For robot pick and place tasks, from a stream of images and raw signals, the edge computer system is tasked to: (1) extract temporal information by semantic filtering over raw data streams, (2) infer on-going events, such as robot approaching, gripping, picking, transporting, releasing, object aligned/misaligned, and feature matched, based on temporal information extracted from sensors and vision feeds, (3) establish domain knowledge graphs by linking extracted information, (4) rapidly query the graph for connected nodes and deriving continuous optimization towards task accomplishment. As a result, task-specific knowledge graphs can be developed to encode large-scale background knowledges of entities, events, data sources, relations, and attributes. This will facilitate a synchronized cognition of working status including equipment, sensors, process flow, material properties, parts structural behaviors, manufacture features, gripping tools, and working principles. As an example, during assembly tasks, combining established domain knowledges of assembly sequences and manufacture features, alternative operation routes can be calculated near real-time based on visual and sensory detection results.

**Figure 16:**
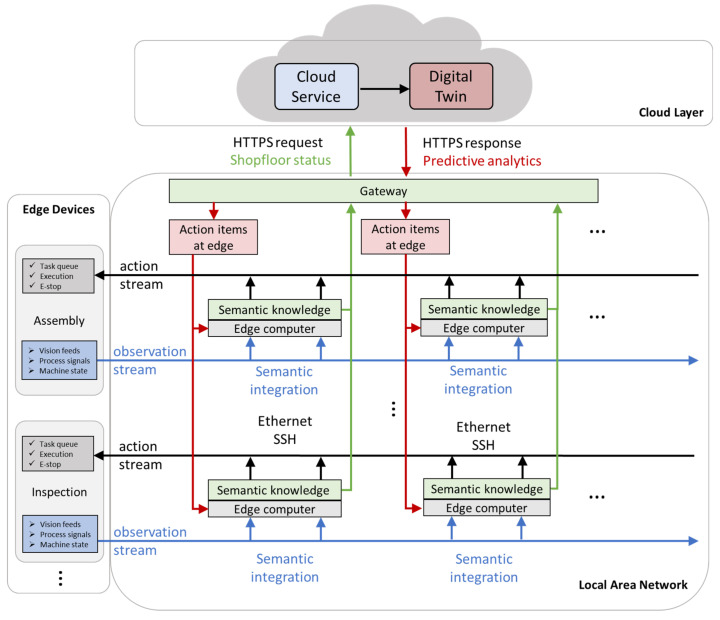
Data flow and implementable communication protocols in proposed decentralized control architecture.

To further apply to enterprise-level management systems, shop-floor digital twin with cognitive capabilities can be deployed over the cloud services so that the overview of the factory statistics can be transmitted to request analytical results and action recommendations. These query results from cognitive digital twin, implemented as knowledge graphs for large-scale systems, will be pushed to the edge computer network for local processing via RestAPI. In Figure 16, we demonstrate the dataflow and implementable protocols to facilitate control decentralization. Three interconnected layers are built to accommodate edge devices, local area network, and cloud layer. Manufacturing equipment communicates with edge computers via Industrial Ethernet and SSH protocols to exchange process signals, vision feeds, and machine state for action items. After semantic integration, knowledge messages are exchanged with cloud predictive twin for analytical results.

## 6. Conclusions

Rapid event-understanding by both machine and humans in highly complex manufacturing scenes needs to be implemented for future cyber-physical manufacturing systems. This work proposes a novel implementation of a vision-aided control system powered by online image inference for rapid adaptive process control. An automated semantic integration system deployable to general signal-based control loops is hereby realized. Proposed system infers manufacturing events from image-based data feeds, and triggers process control signals. Image inference approaches are implemented: (1) cloud-based ML model query and (2) edge end object detection with shape recognition. Depending on use cases and requirements, these two approaches can be designated with different event detection tasks to monitor manufacturing scenes. For instance, shape critical object detection can be easily customized for visual inspection during both additive and subtractive manufacturing, as the defect types are recognizable by detected region shapes. Coupled with conventional industrial sensor signals, proposed systems can rapidly understand process events, able to feed into a manufacturing ontology developed by either expert or ML-enabled cyber systems. Essentially, this work enables a new vision-based communication pipeline to establish an informative connectivity between virtual and physical world. This manages to deploy manufacturing AI enclosed in emerging innovative industrial control loops, that is driven by digital twin, cloud services, and edge computing. To conclude, the authors show that using a machine vision integrated cyber-physical system (CPS) with self-awareness can potentially lead to system self-configuration and semantic human–machine interoperability. System capability of interoperability is significant because it can integrate cyber infrastructure with both data-driven and rule-based intelligence, by which means artificial intelligence and expert knowledge can interact and converge to a joint effort to complex process optimization.

The future work intends to: (1) Further reduce the Mask shape loss to reach a precise shape-critical process event detection. This can be enabled by a precise image annotation method using the digital twin. Section 3.2. (2) Developing a comprehensive manufacturing ontology towards a self-aware and self-configurable CPS; (3) Deployment of computing powers to the field device edge ends. This is accomplished by leveraging Application Specific Integrated Circuit (ASIC) and Field Programmable Gate Array (FPGA) to empower hardware accelerated computing [11]; (4) Connecting manufacturing worlds by semantic representation. To conclude, the goal of subsequent research work is to enable human-in-the-loop machine adaptivity by semantic human–machine interoperation.

## Figures and Tables

**Table 1:** The object detection performance of Mask R-CNN in our system.

Image Count	Mean Average Precision (mAP)	Total Running Time (In seconds)	Running Time Per Image (In Seconds)
10	0.9500	3.7368	0.3737
10	0.9800	3.7609	0.3761
10	0.9620	3.8799	0.3880
10	0.9553	3.9268	0.3927
50	0.9940	20.6661	0.4134
50	0.9458	19.2803	0.3856
50	0.9510	19.8198	0.3964
100	0.9602	39.5330	0.3953
100	0.9760	40.1176	0.4012
100	0.9762	39.4829	0.3948

## Data Availability

Not applicable.
